# Parental willingness and influencing factors for school-based mental health screening in Eastern China

**DOI:** 10.1186/s12889-026-26766-x

**Published:** 2026-03-03

**Authors:** Xiaolian Dong, Xinyue Pang, Yingfeng Chen, Jiangnan Li, Yuyang Xie, Qi Zhao, Zhenguo Zhu, Chaowei Fu, Yue Chen, Na Wang

**Affiliations:** 1 Deqing County Center for Disease Prevention and Control, Huzhou, China; 2 Department of Epidemiology, School of Public Health, Key Laboratory of Public Health Safety, Fudan University, 130 Dongan Road, Xuhui District, Shanghai, 200032 China; 3 Department of Social Medicine, School of Public Health, Fudan University, Shanghai, China; 4 School of Epidemiology and Public Health, Faculty of Medicine, University of Ottawa, Ottawa, ON K1H8M5 Canada

**Keywords:** Mental health, Schools, Screening, Parental Willingness

## Abstract

**Background:**

Adolescent mental health issues are increasingly prominent, making early screening essential for intervention. Schools, as the primary environment for adolescents, serve as ideal settings for screening programs. Parents, as key decision-makers, directly influence participation through their willingness. This study aimed to assess parents’ acceptance of school-based mental health screening among Chinese middle school students, identified relevant factors and concerns, with the goal of providing valuable insights for future programs.

**Methods:**

A cross-sectional survey was conducted in Deqing County, Zhejiang Province, involving parents from three junior high schools. A total of 2,872 valid questionnaires were collected. A self-administered questionnaire assessed sociodemographic characteristics, mental health awareness, screening-related concerns, and willingness. Logistic regression analysis identified associated factors.

**Results:**

89.4% of parents expressed willingness for children to participate in screening. Parents of girls (OR = 0.68, 95% CI: 0.53–0.87) and older parents (age group 45 + vs. < 40 years: OR = 0.71, 95% CI: 0.51–0.99) were less willing, whereas those with high school education (high school/vocational school vs. junior high school or below: OR = 1.54, 95% CI: 1.11–2.13) and higher household income (5000–9999 vs. < 5000 RMB/month: OR = 1.77, 95% CI: 1.29–2.43; 10000 + vs. < 5000 RMB/month: OR = 1.71, 95% CI: 1.20–2.44) showed greater willingness. Parents whose children had a history of mental illness (OR = 0.46, 95% CI: 0.22–0.97) or no prior screening (OR = 0.25, 95% CI: 0.18–0.34) were less willing. Cognitive factors were significant: perceiving mental health issues as unimportant (OR = 0.13, 95% CI: 0.07–0.23), not severe (OR = 0.49, 95% CI: 0.35–0.71) or lacking knowledge (OR = 0.37, 95% CI: 0.24–0.57) was associated with lower willingness, whereas awareness of school-based services was associated with higher willingness (1–2 types: OR = 2.75, 95% CI: 1.86–4.05; ≥3 types: OR = 6.88, 95% CI: 3.29–14.41). Key concerns included children’s comprehension of questions, tool validity, and result reliability. Worries that screening content could negatively influence children were associated with lower willingness (OR = 0.66, 95% CI: 0.45–0.96).

**Conclusions:**

Parents of Chinese junior high school students generally support screening. Willingness is associated with child’s gender, parental age, economic status, mental health awareness and concerns. Enhancing parents’ mental health knowledge and improving tool credibility may be associated with greater parental willingness of children’s mental health screening.

**Supplementary Information:**

The online version contains supplementary material available at 10.1186/s12889-026-26766-x.

## Background

Mental health is an essential component of overall well-being, and adolescence represents a high-risk period for the development of mental health issues. The prevalences of clinically significant generalized anxiety and depressive symptoms among large youth cohorts were approximately 11.6% [1] and 12.9% [2], respectively, posing a significant public health challenge. The COVID-19 pandemic has intensified mental health issues among children and adolescents, as a meta-analysis [3] incorporating 29 studies estimated significantly higher proportions for anxiety (20.5%) and depression (25.2%) symptoms during the pandemic. Global Burden of Disease Study [4] found that the age-standardized prevalence of mental disorders in children and adolescents was 8.9% in China in 2021. The China Youth Development Report (2020) [5] revealed that approximately 30 million children and adolescents under the age of 17 in China were affected with various emotional and behavioral issues. Mental health problems in adolescents can lead to serious consequences, including social dysfunction [6], academic difficulties [7], and increasing risk of self-harm and suicide [8]. Without timely and effective intervention, these issues may persist into adulthood, imposing long-term burdens on families and society [9]. With rapid socioeconomic development and the widespread use of electronic devices and social media [10], adolescent mental health problems have become increasingly prominent.

Mental health screening is a crucial tool for identifying potential psychological issues. It facilitates the early detection and treatment of mental health concerns, helping to prevent the progression of disorders and reduce long-term harm [11], particularly for common adolescent conditions such as depression and anxiety [12]. Given that adolescents spend most of their time in school, school-based screening is often more convenient [13] and more acceptable to students [14] compared to screenings conducted in hospitals or other professional settings. As a proactive and preventive approach, universal school mental health screening ensures regular assessment for all students [15]. Previous research has indicated that universal school-based mental health screening plays a vital role in adolescent mental health interventions [16, 17]. This approach promotes equitable access to mental health services, effectively bridges regional healthcare disparities and individual socioeconomic differences [18], and helps protect the mental health of vulnerable populations.

Parents play a critical role as decision-makers in adolescents’ access to mental health services, with their attitudes fundamentally determining children’s participation in school-based screening programs [11]. However, few studies have explored parents’ willingness to allow their children to participate in such screenings. Previous research suggests that parents generally hold positive attitudes, with factors such as demographic characteristics, awareness of mental health, and perceptions of screening influencing their willingness [19, 20]; however, findings have been inconsistent. Moreover, most studies have focused on screenings for specific mental disorders such as depression, rather than comprehensive multidimensional mental health assessments. Additionally, most of these studies have been conducted in European and North American countries [19–21], whereas evidence on Chinese parents’ attitudes toward school-based screening remains limited and warrants further investigation. The most recent study [22] explored Chinese parents’ willingness to participate in universal school-based depression screening, reporting that 92.4% supported the program and that greater knowledge about depression was associated with higher acceptance. However, it may not have fully accounted for factors such as history of psychological disorders, previous screening experiences, or the parent–child relationship. Given cultural and contextual differences, and the fact that schools in China often conduct general mental health screenings rather than screenings targeting specific mental problems, further research is needed to explore the factors that influence Chinese parents’ willingness to support such screening programs.

In recent years, China has placed increasing emphasis on adolescent mental health, introducing policies recommending that county-level education departments conduct mental health assessments at least once per academic year for students in upper primary, middle, and high schools [23]. The junior high school years (approximately ages 12–15) represent a critical period for adolescents’ psychological development and personality formation. Studies have shown that Chinese junior high school students face significant mental health challenges [24–26]. Therefore, this study aims to investigate willingness of parents of Chinese junior high school students to allow their children to participate in school-based mental health screening, and to explore how sociodemographic characteristics, awareness levels, and screening-related concerns influence that willingness. The findings are intended to support the advancement of school-based screening initiatives and contribute to the improvement of adolescent mental health.

## Method

### Participants

This cross-sectional survey was conducted in April 2025, in Deqing County, Zhejiang Province, located in eastern China, where education and mental health policy environments are consistent across regions. Three junior high schools (grades 7–9) were selected from the eastern, central, and western regions of the county, respectively. Within each selected school, half of the classes from each grade were randomly selected. Online self-reported questionnaires were distributed to parents through the Wenjuanxing platform (https://www.wjx.cn). A total of 2,456 students were included, with the participation of at least one parent per student required, although both parents were allowed to complete the questionnaire. To address privacy concerns and to elicit more genuine attitudes and intentions, the survey was conducted anonymously.

**Fig. 1:**
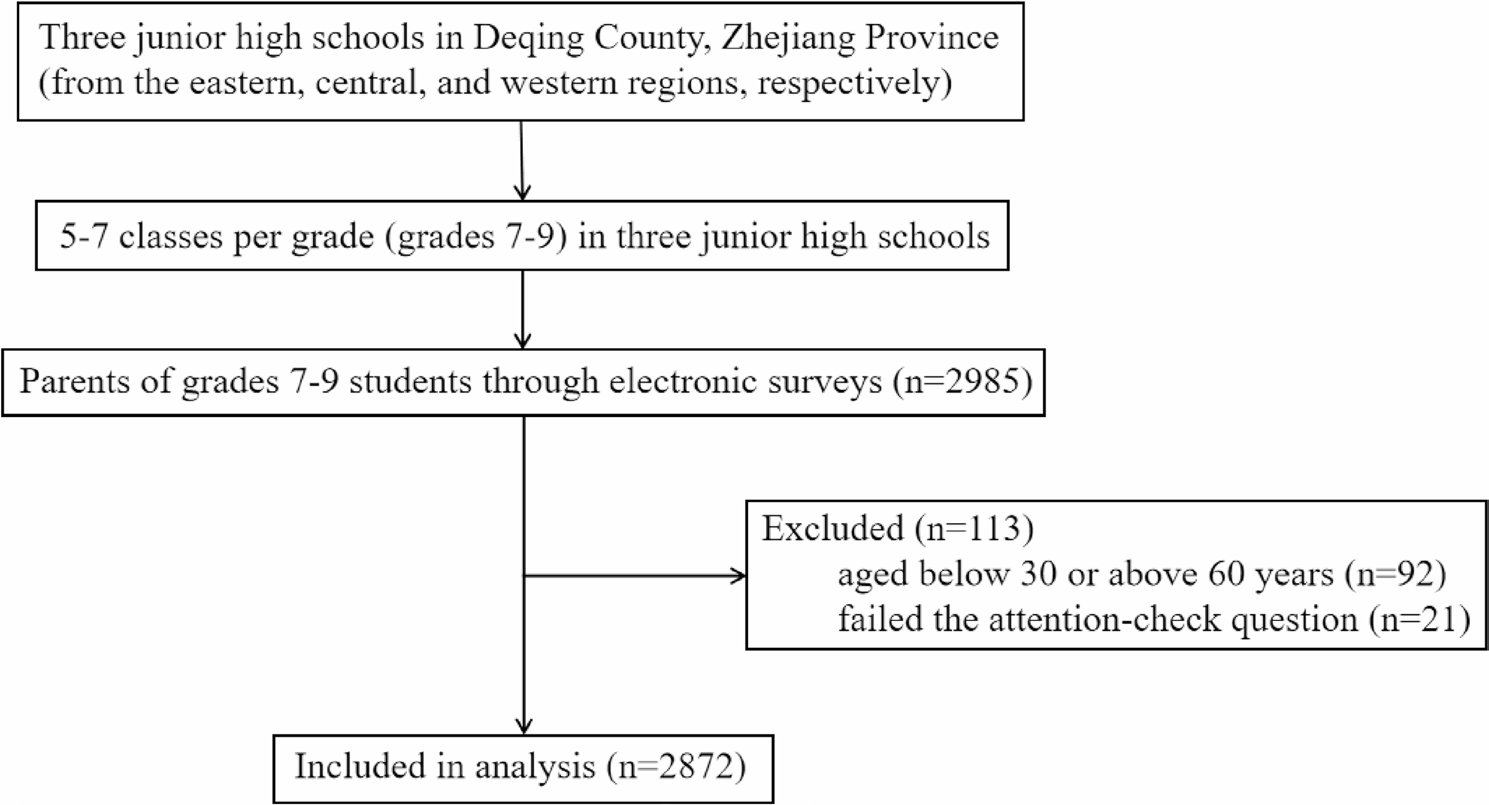
Screening process for participants

A total of 2985 questionnaires were collected. Informed consent was obtained from all participating parents. After excluding outliers (parents aged below 30 or above 60 years) and responses that failed the attention-check question (“Which city is the capital of China: Beijing, Shanghai or Guangzhou?”), 2872 valid responses were included in the analysis, with a validity rate of 96.2%. The selection process of study participants is presented in Fig. 1.

### Study design and measures

The questionnaire was self-administered. A general reliability estimate was not calculated, as the questionnaire consisted of independent items without a common underlying construct or total scale score. The self-designed questionnaire aimed to assess parents’ willingness to allow their children to participate in school-based mental health screening and to examine three main aspects (sociodemographic characteristics, perspective on mental health, and screening-related concerns) that may affect parents’ willingness (supplementary file 1).

Participants were asked to rate their willingness to support school-based mental health screening on a 5-point Likert-type question (from strongly unwilling [1] to strongly willing [5]). Responses of “strongly willing” and “somewhat willing” were categorized as willing, while “uncertain” “somewhat unwilling” and “strongly unwilling” were categorized as hesitating or unwilling. Six items assessed participants’ awareness of adolescent mental health issues (importance of mental health, severity of mental health issues and knowledge of mental health) and their understanding of their own child’s mental health well-being (psychological status, emotional or physiological abnormalities and school mental health services). Eleven items assessed participants’ concerns about mental health screening, based on issues identified in previous studies [19, 22] (including not understanding the screening questions, and screening tools lacking scientific validity, etc.). Participants rated their agreement with each concern on 5-point Likert-type questions (from strongly disagree [1] to strongly agree [5]). Responses of “strongly agree” “somewhat agree” and “neutral” were categorized as indicating agreement with each concern.

Sociodemographic information about both parents and children was also collected. Participants provided information on their own gender, age, education level, household income and region of residence, as well as their child’s gender, grade, academic performance, history of psychological disorders and prior experience of school-based mental health screening. The parent-child relationship was assessed through questions on family structure, daily communication, parenting style and a self-evaluation of relationship quality. Family structure was categorized as intact (living with both parents), single-parent, stepfamily, and left-behind (parents migrating for work). Parenting style was measured using a single self-report item and categorized into five types: authoritarian, authoritative, permissive, indulgent, and unspecified [27].

### Statistical analysis

Statistical analyses were performed using R v.4.3.2. Qualitative variables were reported as frequencies and percentages. Willingness to allow children to participate in school-based mental health screening was dichotomized as follows: responses of “strongly willing” and “somewhat willing” were categorized as indicating willingness, while “uncertain” “somewhat unwilling” and “strongly unwilling” responses were categorized as hesitating or unwilling. Concerns about screening were assessed by categorizing responses of “strongly agree” “somewhat agree” and “neutral” as indicative of agreement with each concern.

Descriptive statistics were used to assess the sociodemographic characteristics of the participants. Differences of basic characteristics between parents who were willing to allow children to participate in school-based mental health screening and those who were not were compared using the chi-square test.

Both univariate and multivariate logistic regression analyses were performed to examine the sociodemographic factors influencing parental willingness, and crude and adjusted odds ratios (ORs) and their 95% confidence intervals (95% CIs) were calculated for the associations. Variables that showed statistical significance in the univariate analysis were included in the multivariate model using stepwise regression. Multivariate Model 1 included both the child’s and the parent’s gender, whereas Multivariate Model 2 focused on the parent-child relationship, which was constructed by combining child’s and the parent’s gender. Logistic regression models were also used to explore the influence of factors related to adolescents’ mental health cognition and various concerns about the screening on parents’ willingness. Model 1 included only the specific cognitive or concern-related factor, and Model 2 adjusted for sociodemographic variables. A two-tailed p-value < 0.05 was considered statistical significance in all analyses.

## Results

### Characteristics of participants

Of the 2872 participants, 1716 (59.7%) were mothers, with a mean (standard deviation, SD) age of 41.1 (4.9) years. Among their children, 1465 (51.0%) were boys with a balanced distribution across the three grades. More than half of the parents (55.4%) lived in urban areas, and the vast majority of children lived with their parents. A total of 63 children (2.2%) had been diagnosed with mental illness, and 1,329 (46.3%) had undergone mental health screening at school.

Significant differences between parents who were willing to have their children participate in school-based mental health screening and those who were unwilling or hesitant, were observed by parental gender (*P* = 0.025), age (*P* = 0.018), child’s gender (*P* = 0.017), educational attainment (*P* = 0.001), family income (*P* < 0.001), frequency of parent-child communication (*P* = 0.017), parenting style (*P* < 0.001) and previous experiences of school-based mental health screening (*P* < 0.001). Detailed information on participant characteristics is presented in Table 1.

**Table 1:** Sociodemographic characteristics of participants [n (%)] by willingness of school-based mental health screening for children

Characteristics	Total (*n* = 2872)	Willingness toward screening (*n* = 2569)	Unwillingness or hesitancy toward screening (*n* = 303)	χ^2^ value	*P* value
Child’s gender				5.65	0.017
Male	1465 (51.0)	1330 (90.8)	135 (9.2)		
Female	1407 (49.0)	1239 (88.1)	168 (11.9)		
Child’s grade				1.18	0.554
Grade 7	930 (32.4)	824 (88.6)	106 (11.4)		
Grade 8	981 (34.2)	879 (89.6)	102 (10.4)		
Grade 9	961 (33.5)	866 (90.1)	95 (9.9)		
Parent				4.99	0.025
Father	1156 (40.3)	1016 (87.9)	140 (12.1)		
Mother	1716 (59.7)	1553 (90.5)	163 (9.5)		
Type of parent-child relationship				15.88	0.001
Father-son	619 (21.6)	561 (90.6)	58 (9.4)		
Mother-son	846 (29.5)	769 (90.9)	77 (9.1)		
Father-daughter	537 (18.7)	455 (84.7)	82 (15.3)		
Mother-daughter	870 (30.3)	784 (90.1)	86 (9.9)		
Parent’s age (years)				8.05	0.018
<40	1297 (45.2)	1180 (91.0)	117 (9.0)		
40–44	1013 (35.3)	902 (89.0)	111 (11.0)		
45+	562 (19.6)	487 (86.7)	75 (13.3)		
Parent’s education level				13.10	0.001
Junior high school or below	1128 (39.3)	986 (87.4)	142 (12.6)		
High school/vocational school	869 (30.0)	803 (92.4)	66 (7.6)		
College/bachelor’s degree or above	875 (30.5)	780 (89.1)	95 (10.9)		
Region of residence				0.02	0.889
Urban	1591 (55.4)	1422 (89.4)	169 (10.6)		
Rural	1281 (44.6)	1147 (89.5)	134 (10.5)		
Family monthly income (RMB)				19.25	<0.001
<5000	515 (17.9)	433 (84.1)	82 (15.9)		
5000–9999	1394 (48.5)	1265 (90.7)	129 (9.3)		
10,000+	963 (33.5)	871 (90.4)	92 (9.6)		
Family structure				1.67	0.643
Intact family	2551 (88.8)	2282 (89.5)	269 (10.5)		
Single-parent family	172 (6.0)	157 (91.3)	15 (8.7)		
Stepfamily	94 (3.3)	83 (88.3)	11 (11.7)		
Left-behind family	55 (1.9)	47 (85.5)	8 (14.5)		
Daily communication with child				10.25	0.017
Every day	2110 (73.5)	1902 (90.1)	208 (9.9)		
4–6 times/week	191 (6.7)	176 (92.1)	15 (7.9)		
1–3 times/week	450 (15.7)	389 (86.4)	61 (13.6)		
Never	121 (4.2)	102 (84.3)	19 (15.7)		
Parenting style				31.35	<0.001
Authoritative	862 (30.0)	789 (91.5)	73 (8.5)		
Authoritarian	184 (6.4)	163 (88.6)	21 (11.4)		
Permissive	1020 (35.5)	935 (91.7)	85 (8.3)		
Indulgent	231 (8.0)	201 (87.0)	30 (13.0)		
Uncertain	575 (20.0)	481 (83.7)	94 (16.3)		
Parent-child relationship				4.12	0.127
Very harmonious	851 (29.6)	767 (90.1)	84 (9.9)		
Fairly harmonious	1507 (52.5)	1355 (89.9)	152 (10.1)		
Average/strained​	514 (17.9)	447 (87.0)	67 (13.0)		
Child’s academic performance				6.40	0.094
Top tier	714 (24.9)	655 (91.7)	59 (8.3)		
Upper middle tier	1065 (37.1)	941 (88.4)	124 (11.6)		
Lower middle tier	724 (25.2)	640 (88.4)	84 (11.6)		
Bottom tier	369 (12.8)	333 (90.2)	36 (9.8)		
History of psychological disorders				4.19	0.123
No	2685 (93.5)	2410 (89.8)	275 (10.2)		
Yes	63 (2.2)	54 (85.7)	9 (14.3)		
Unsure	124 (4.3)	105 (84.7)	19 (15.3)		
History of school-based mental health screening				88.49	<0.001
Yes	1329 (46.3)	1266 (95.3)	63 (4.7)		
No	851 (29.6)	718 (84.4)	133 (15.6)		
Unsure	692 (24.1)	585 (84.5)	107 (15.5)		

### Parental willingness to allow their children to participate in school-based mental health screening

Overall, the majority of parents (*n* = 2569, 89.4%) were either strongly willing or somewhat willing to have their children participate in school-based mental health screening. A small proportion of parents (*n* = 73, 2.5%) were somewhat unwilling or strongly unwilling, while a sizeable proportion of parents (*n* = 230, 8.0%) reported an uncertain attitude.

### Sociodemographic factors associated with willingness to allow their children to participate in school-based mental health screening

In the univariate analysis, several factors showed significant associations with parents’ willingness for their children to participate in mental health screening, including parents’ gender, age, educational level, and household income, as well as children’ s gender, academic performance, and screening history, along with communication frequency and parenting style.

Multivariable logistic regression analysis showed that children’s gender, parents’ age and education level, family monthly income, history of psychological disorders and prior school-based mental health screening were significant factors influencing parental willingness (Table 2). Compared to parents of boys (Model 1), parents of girls were less willing to have their children undergo school-based mental health screening (OR = 0.68, 95% CI: 0.53–0.87, *P* = 0.002). Compared to parents under the age of 40, parents aged 45 or more showed lower willingness (OR = 0.71, 95% CI: 0.51–0.99, *P* = 0.045). Parents with higher educational level were more likely to participate than those with junior high school education or below (OR = 1.54, 95% CI: 1.11–2.13, *P* = 0.009). Parents with family monthly income 5000 RMB or more showed greater willingness than those earning less than 5000 RMB (5000–9999 RMB: OR = 1.77, 95% CI: 1.29–2.43, *P* < 0.001; 10000 + RMB: OR = 1.71, 95% CI: 1.20–2.44, *P* = 0.003). In addition, parents whose children had a history of psychological disorders (OR = 0.46, 95% CI: 0.22–0.97, *P* = 0.040), as well as whose children who had not participated in (OR = 0.25, 95% CI: 0.18–0.34, *P* < 0.001) or were unsure about participation in previous school-based mental health screening (OR = 0.28, 95% CI: 0.20–0.39, *P* < 0.001) were less willing to have their children undergo such screening. When considering to parent-child relationships (Model 2), fathers of daughters were less willing to have children participate in the screening compared to fathers of sons (OR = 0.52, 95% CI: 0.36–0.76, *P* < 0.001).

**Table 2:** Sociodemographic factors associated with parental willingness of school-based mental health screening for children

Characteristics	Univariate Model		Multivariate-Model 1		Multivariate-Model 2	
	OR (95% CI)	*P* value	OR (95% CI)	*P* value	OR (95% CI)	*P* value
Child’s gender						
Male	1.00		1.00			
Female	0.75 (0.59,0.95)	0.018	0.68 (0.53,0.87)	0.002		
Child’s grade						
Grade 7	1.00					
Grade 8	1.11 (0.83,1.48)	0.483				
Grade 9	1.17 (0.87,1.57)	0.286				
Parent						
Father	1.00		1.00			
Mother	1.31 (1.03,1.67)	0.026	1.23 (0.95,1.60)	0.113		
Type of parent-child relationship						
Father-son	1.00				1.00	
Mother-son	1.03 (0.72,1.48)	0.861			0.96 (0.66,1.40)	0.828
Father-daughter	0.57 (0.40,0.82)	0.002			0.52 (0.36,0.76)	<0.001
Mother-daughter	0.94 (0.66,1.34)	0.740			0.80 (0.55,1.16)	0.237
Parent’s age (years)						
<40	1.00		1.00		1.00	
40–44	0.81 (0.61,1.06)	0.122	0.82 (0.61,1.10)	0.187	0.82 (0.61,1.09)	0.175
45+	0.64 (0.47,0.88)	0.005	0.71 (0.51,0.99)	0.045	0.72 (0.52,1.01)	0.051
Parent’s education level						
Junior high school or below	1.00		1.00		1.00	
High school/vocational school	1.75 (1.29,2.38)	<0.001	1.54 (1.11,2.13)	0.009	1.56 (1.13,2.16)	0.007
College/bachelor’s degree or above	1.18 (0.90,1.56)	0.234	0.95 (0.70,1.30)	0.768	0.96 (0.70,1.31)	0.785
Region of residence						
Urban	1.00					
Rural	1.02 (0.80,1.29)	0.889				
Family monthly income (RMB)						
<5000	1.00		1.00		1.00	
5000–9999	1.86 (1.38,2.50)	<0.001	1.77 (1.29,2.43)	<0.001	1.78 (1.30,2.44)	<0.001
10,000+	1.79 (1.30,2.47)	<0.001	1.71 (1.20,2.44)	0.003	1.73 (1.21,2.46)	0.003
Family structure						
Intact family	1.00					
Single-parent family	1.23 (0.72,2.13)	0.449				
Stepfamily	0.89 (0.47,1.69)	0.720				
Left-behind family	0.69 (0.32,1.48)	0.344				
Daily communication with child						
Everyday	1.00					
4–6 times/week	1.28 (0.74,2.22)	0.371				
1–3 times/week	0.70 (0.51,0.95)	0.021				
Never	0.59 (0.35,0.98)	0.041				
Parenting style						
Authoritative	1.00		1.00		1.00	
Authoritarian	0.72 (0.43,1.20)	0.207	0.85 (0.50,1.45)	0.555	0.85 (0.50,1.45)	0.554
Permissive	1.02 (0.73,1.41)	0.916	1.15 (0.82,1.61)	0.412	1.15 (0.82,1.61)	0.417
Indulgent	0.62 (0.39,0.97)	0.038	0.74 (0.46,1.18)	0.202	0.74 (0.46,1.19)	0.213
Uncertain	0.47 (0.34,0.66)	<0.001	0.58 (0.41,0.82)	0.002	0.58 (0.41,0.82)	0.002
Parent-child relationship						
Very harmonious	1.00					
Fairly harmonious	0.98 (0.74,1.29)	0.867				
Average/strained​	0.73 (0.52,1.03)	0.072				
Child’s academic performance						
Top tier	1.00					
Upper middle tier	0.68 (0.49,0.95)	0.022				
Lower middle tier	0.69 (0.48,0.97)	0.035				
Bottom tier	0.83 (0.54,1.29)	0.411				
History of psychological disorders						
No	1.00		1.00		1.00	
Yes	0.68 (0.33,1.40)	0.300	0.46 (0.22,0.97)	0.040	0.45 (0.21,0.95)	0.037
Unsure	0.63 (0.38,1.04)	0.073	0.79 (0.46,1.35)	0.382	0.78 (0.46,1.34)	0.374
History of school-based mental health screening						
Yes	1.00		1.00		1.00	
No	0.27 (0.20,0.37)	<0.001	0.25 (0.18,0.34)	<0.001	0.24 (0.18,0.34)	<0.001
Unsure	0.27 (0.20,0.38)	<0.001	0.28 (0.20,0.39)	<0.001	0.28 (0.20,0.39)	<0.001

Multivariate-Model 1: Included child's gender, parent’s gender, parent's age, parent's education level, family monthly income (RMB), parenting style, history of psychological disorders, and history of school-based mental health screening

Multivariate-Model 2: Included type of parent-child relationship, parent's age, parent's education level, family monthly income (RMB), parenting style, history of psychological disorders, and history of school-based mental health screening

### Cognitive factors associated with parental willingness for school-based mental health screening

As shown in Table 3, parents’ perceptions of mental health issues were significantly associated with their willingness to have their children participate in screening. Parents who were unaware of the importance of adolescents’ mental health (quite important: OR = 0.40, 95% CI: 0.29–0.55, *P* < 0.001; neutral/not important: OR = 0.13, 95% CI: 0.07–0.23, *P* < 0.001) and the severity of mental health issues (neutral/not severe: OR = 0.49, 95% CI: 0.35–0.71, *P* < 0.001; unaware: OR = 0.38, 95% CI: 0.20–0.72, *P* = 0.002), as well as those who did not pay attention to mental health knowledge (OR = 0.37, 95% CI: 0.24–0.57, *P* < 0.001), had lower odds of willingness. Conversely, The greater the variety the mental health services offered by school, the higher the parental willingness for their children undergo screening (1–2 types: OR = 2.75, 95% CI: 1.86–4.05, *P* < 0.001; ≥3 types: OR = 6.88, 95% CI: 3.29–14.41, *P* < 0.001). Both among fathers and mothers, these mental health awareness factors were significantly associated with screening willingness. No significant interaction effects were found between parent gender and mental health awareness factors.

**Table 3:** Cognitive factors associated with parental willingness of school-based mental health screening for children

Characteristics	Univariate Model		Multivariate Model	
	OR (95% CI)	*P* value	OR (95% CI)	*P* value
Importance of mental health to adolescents​				
Extremely important	1.00		1.00	
Quite important	0.38 (0.28, 0.51)	<0.001	0.40 (0.29, 0.55)	<0.001
Neutral/not important	0.13 (0.08, 0.21)	<0.001	0.13 (0.07, 0.23)	<0.001
Severity of adolescent mental health issues​				
Extremely severe	1.00		1.00	
Quite severe	0.82 (0.59, 1.14)	0.234	0.81 (0.58, 1.14)	0.220
Neutral/not severe	0.52 (0.37, 0.73)	<0.001	0.49 (0.35, 0.71)	<0.001
Unaware	0.32 (0.17, 0.59)	0.003	0.38 (0.20, 0.72)	0.002
Understanding of child’s psychological status​				
Very knowledgeable	1.00		1.00	
Fairly knowledgeable	0.86 (0.60, 1.23)	0.406	0.98 (0.67, 1.43)	0.918
Average	0.64 (0.43, 0.95)	0.028	0.81 (0.53, 1.24)	0.336
Limited knowledge/unaware	0.82 (0.43, 1.55)	0.533	1.31 (0.66, 2.61)	0.438
Emotional or physiological abnormalities in child (past 3 months)​				
None	1.00		1.00	
1–2 types	0.85 (0.64, 1.11)	0.237	0.88 (0.66, 1.18)	0.404
≥ 3 types	1.03 (0.73, 1.45)	0.880	1.16 (0.81, 1.67)	0.418
School mental health services				
None	1.00		1.00	
1–2 types	3.77 (2.62, 5.43)	<0.001	2.75 (1.86, 4.05)	<0.001
≥ 3 types	11.02 (5.41, 22.44)	<0.001	6.88 (3.29, 14.41)	<0.001
Unclear	1.36 (0.99, 1.86)	0.055	1.27 (0.90, 1.77)	0.169
Knowledge of mental health				
Yes	1.00		1.00	
Unaware or unconcerned	0.28 (0.19, 0.43)	<0.001	0.37 (0.24, 0.57)	<0.001

Multivariate-Model: Adjusted for child's gender, parent’s gender, parent's age, parent's education level, family monthly income (RMB), parenting style, history of psychological disorders, and history of school-based mental health screening

### Concerns associated with parental willingness for school-based mental health screening

Table 4 shows participants’ concerns for school-based mental health screening, ranked from highest to lowest agreement. The agreement rate for parental concerns ranged from 71.7% to 90.5%. The most commonly endorsed concerns included: children may not understand screening questions (90.5%), a lack of scientific validity and accuracy in the screening tools (90.1%), and the possibility that screening may not accurately reflect the children’s mental health state (90.1%).

**Table 4:** Concerns associated with parental willingness of school-based mental health screening for children

Concerns	Total (*n* = 2872)	Willingness toward screening (*n* = 2569)	Unwillingness or hesitancy toward screening (*n* = 303)	Univariate Model		Multivariate Model	
				OR (95% CI)	*P* value	OR (95% CI)	*P* value
Children may not understand screening questions	2599 (90.5)	2326 (90.5)	273 (90.1)	1.05 (0.71,1.57)	0.804	1.36 (0.89,2.08)	0.149
Insufficient scientific validity and accuracy of screening tools	2589 (90.1)	2312 (90.0)	277 (91.4)	0.84 (0.55,1.29)	0.432	0.89 (0.57,1.39)	0.617
Screening may not reflect true psychological status	2587 (90.1)	2316 (90.2)	271 (89.4)	1.08 (0.73,1.59)	0.695	1.17 (0.78,1.76)	0.447
Screening process may cause discomfort to children	2541 (88.5)	2279 (88.7)	262 (86.5)	1.23 (0.87,1.75)	0.248	1.37 (0.94,1.99)	0.099
Schools may lack professional screening capacity	2453 (85.4)	2179 (84.8)	274 (90.4)	0.59 (0.40,0.88)	0.010	0.67 (0.45,1.01)	0.059
Questionnaire content may implicitly influence children	2357 (82.1)	2090 (81.4)	267 (88.1)	0.59 (0.41,0.84)	0.004	0.66 (0.45,0.96)	0.028
Lack of post-screening support	2332 (81.2)	2081 (81.0)	251 (82.8)	0.88 (0.65,1.21)	0.440	0.88 (0.63,1.23)	0.459
Potential costs associated with screening and interventions	2270 (79.0)	2026 (78.9)	244 (80.5)	0.90 (0.67,1.22)	0.501	0.96 (0.70,1.32)	0.811
Risk of privacy breaches during screening	2173 (75.7)	1928 (75.0)	245 (80.9)	0.71 (0.53,0.96)	0.026	0.76 (0.55,1.03)	0.080
Screening may take time away from academic studies	2099 (73.1)	1861 (72.4)	238 (78.5)	0.72 (0.54,0.96)	0.024	0.88 (0.65,1.19)	0.408
Risk of labeling or stigmatization	2058 (71.7)	1827 (71.1)	231 (76.2)	0.77 (0.58,1.01)	0.062	0.78 (0.58,1.04)	0.088

Multivariate-Model: Adjusted for child's gender, parent’s gender, parent's age, parent's education level, family monthly income (RMB), parenting style, history of psychological disorders, and history of school-based mental health screening

These concerns were also associated with parents’ willingness to allowed their children to participate in the screening. Specifically, Parents who expressed greater concern about the potential impact of questionnaire content on their children (OR = 0.66, 95% CI: 0.45–0.96, *P* = 0.028) were significantly less willing to support participation.

## Discussion

This study found that approximately 89.4% of parents were willing to allow their children to participate in school-based mental health screening. Lower willingness was observed among parents of girls, older parents, those with lower education levels, and those with lower household incomes, as well as parents whose children had a history of mental illness or had not previously participated in school-based screenings. Inadequate awareness of the severity of adolescent mental health issues and limited knowledge of school-based mental health services were also associated with reduced willingness. Parents who were unwilling or hesitant to participate expressed greater multidimensional concerns about the screening.

Parents of Chinese junior high school students generally supported school-based mental health screening, consistent with previous findings. A multinational survey [20] across 19 countries showed that 92.1% of parents (mean child age = 11.1 ± 4.3 years) favored regular mental health screening for their children, although they preferred it conducted in healthcare settings with the involvement of physicians or psychologists. Most school-based screening studies have focused on depression. A U.S. study [21] reported 70.5% of middle and high school parents considered universal school depression screening appropriate, slightly lower than our findings, potentially due to the sensitive nature of depression. However, a recent Chinese study [22] found that 92.4% of primary and secondary school parents supported universal depression screenings in schools. This discrepancy may stem from differences in the age of the children studied and cultural contexts.

The study identified sociodemographic characteristics of both children and parents as significant factors associated with parental willingness. Parents of girls, particularly in father-daughter relationships, demonstrated lower willingness compared to those in father-son relationships. However, multiple studies have shown that girls are more prone to mental health issues and depressive symptoms than boys [28, 29]. This gender-based willingness discrepancy, which has not been reported in prior research, may be associated with fathers relatively lower understanding of daughters’ psychological status (*P* = 0.003). Foster et al. [30] found that parents of adolescent girls were less likely to consider mental health information important compared to parents of boys (53% vs. 70%, *P* = 0.01). This difference may also reflect protective concerns for adolescent girls’ heightened psychological sensitivity, aimed at preventing potential emotional distress [31]. Older parents exhibited lower willingness, potentially due to traditional notion of focusing solely on physical health and limited awareness of mental health services [32]. In this study, parents aged 45 or older showed lower recognition of the importance (*P* < 0.001) and severity (*P* = 0.002) of adolescent mental health issues. Regarding education effects, existing studies report inconsistent findings. We found parents with a high school education showed higher willingness than those with a junior high education, but no significant difference was observed between junior high school and college-educated parents. Less-educated parents may have a limited understanding of mental health importance and severity (both *P* < 0.001), while highly educated parents may perceive greater self-efficacy in addressing children’s psychological abnormalities independently, potentially relying less on school-based interventions. This finding is supported by a U.S. study on parents’ attitudes toward school-based depression screening for adolescents [13]. Economic status was also significantly associated with willingness, potentially reflecting concerns about mental health service accessibility and the costs of follow-up [33, 34]. Surprisingly, parents whose children had a history of mental illness exhibited significantly lower willingness than those without such a history. Research suggests that the stigma associated with mental illness affects parents’ responses to adolescent mental health issues [35], as they may fear labeling and discrimination if schools become aware of their children’s conditions. Conversely, parents with prior experience in school-based screenings showed higher willingness, suggesting that positive past experiences are related to higher participation rates.

Cognitive factors related to mental health were also associated with parental willingness. Parents who underestimated the importance and severity of adolescent mental health issues or showed little interest in mental health knowledge exhibited significantly lower willingness to allow their children to participate. Research suggests that inadequate recognition of mental health problems is a barrier preventing at-risk adolescents from accessing services [36]. Such parents may overlook the prevalence and severity of adolescent mental health issues, underestimating the importance of early intervention. Fox et al. [13] found that parents with greater knowledge of depression and suicide were more likely to support school-based depression screening and suicide education (depression screening: OR = 8.48, 95% CI: 1.30-55.21, *P* = 0.025; suicide education: OR = 7.99, 95% CI: 1.02–62.68, *P* = 0.048). Conversely, parents who were aware of school mental health services showed significantly higher willingness than those who were unaware, potentially due to greater trust in school mental health programs and confidence in subsequent service support [15]. Notably, 36.4% of parents were unsure whether their schools provided mental health services, highlighting a lack of awareness about how schools handle screening results and the availability of mental health resources. This underscores the need for schools to strengthen communication in this area.

The findings suggest that despite some concerns, most Chinese parents accept school-based universal mental health screenings for their children. The accuracy of screening results was the primary concern among participants, with parents worrying about their children’s ability to understand questions, the scientific validity of screening tools, and whether results would accurately reflect their children’s true psychological state. Similar concerns were reported in a qualitative study of UK primary school parents [19], where parents expressed doubts about the detectability of adolescent mental health issues and the potential for schools to misinterpret responses, leading to false positive or false negative results. A study on universal depression screenings in schools [22] also identified accuracy as the most frequently cited concern among Chinese parents. However, research on screening accuracy remains limited. Some studies suggest that school-based mental health screenings have high false-positive rates [37], such as 20% for social anxiety disorder screenings [38]. Yet, Scott et al. [39] found that school-based screenings had higher sensitivity than assessments conducted by school professionals, improving the identification of students at high risk of suicide and playing an irreplaceable role. Therefore, further research and practical improvement in school-based screening questionnaire are needed to improve screening sensitivity and specificity, thereby reducing unnecessary interventions and minimizing panic.

Additionally, multivariate analysis indicated that concern about the potential negative implications of questionnaire content for children was associated with decreased willingness of screening. Universal screenings for depression and anxiety without adequate supervision and support could potentially trigger distress by heightening emotional awareness [40], although multiple studies have found no adverse psychological effects from such screenings [41, 42]. Mental health education for educators and carefully managing the identification and feedback processes are essential to mitigate potential harms [43]. Future research and practice should focus on refining screening protocols to address parental concerns and convert hesitant or unwilling parents into supporters.

This study, focusing on the school-based mental health screening advocated by Chinese policies, comprehensively examined sociodemographic characteristics, perspective on mental health, and concerns of screening, while also incorporating variables such as children’s screening and psychological disorders history, parenting style, and parent–child communication which have been rarely addressed in previous studies. The findings provide new and valuable insights into the willingness of parents of Chinese junior high school students to allow their children to participate in school-based mental health screening, as well as the factors associated with their attitudes.

### Limitations

This study has several limitations. First, as a cross-sectional study, it cannot establish causal relationships due to the lack of temporal sequencing. Second, since the study was conducted in a county in Zhejiang Province, the findings may not be nationally representative and require validation in the eastern region of China. Third, although the study considered multiple factors, some other potential influencing factors may have been overlooked, necessitating further investigation. Fourthly, the study examined cases where both parents of the same student participated in the survey, which may introduce non-independence of responses.

## Conclusion

This study found that despite concerns, most parents of Chinese junior high school students are willing to allow their children to participate in school-based mental health screenings. Parental willingness is associated with multiple factors, including the child’s gender, parental education level, household income, the child’s history of mental illness and prior screening experiences, as well as parental awareness of mental health. Enhancing parental knowledge of mental health and the importance of screening, improving school-parent communication, and establishing scientific standardized screening mechanisms may be associated with greater parental willingness to have their children participate in mental health screening. The findings of this study provided preliminary evidence that can help identify potential barriers and facilitators for future implementation efforts.

## Supplementary Information

Supplementary Material 1.

Supplementary Material 2.

## Data Availability

The datasets generated and analyzed during the current study are not publicly available due to sensitive information but are available from the corresponding author on reasonable request.
