# Supplementary material for: Parental willingness and influencing factors for school-based mental health screening in Eastern China
Source: BMC Public Health. 2026 Mar 3;26:1157. doi: 10.1186/s12889-026-26766-x (PMC13064065; doi:10.1186/s12889-026-26766-x)
Supplement: Supplementary file 1 — Supplementary Material 1. [file 12889_2026_26766_MOESM1_ESM.docx]

**Parental Willingness and Influencing Factors for School-Based Mental Health Screening in Eastern China**

**Methodological details**

**Pilot Testing:**

A pre-survey was conducted to test the comprehensibility of the questionnaire, and revisions were made based on participant feedback.

**Timing and Data Collection:**

The survey was conducted in April 2025, with data collection completed within 20 days. Links to the electronic questionnaire (through the Wenjuanxing platform) were distributed by teachers separately via WeChat groups of the specific classes selected from the three schools located in the eastern, central, and western regions.

**Quality Control:**

Several quality control measures were implemented during distribution: the questionnaire was restricted to responses from logged-in WeChat accounts, with each account allowed only to a single submission, and it was configured to be non-indexable by search engines. To assess data quality, an attention-check question (“Which city is the capital of China: Beijing, Shanghai or Guangzhou?”) was included, and only responses answering it correctly were retained. Additionally, responses reporting parent ages outside a plausible range (below 30 or above 60 years) were excluded.
