# Supplementary material for: Parental willingness and influencing factors for school-based mental health screening in Eastern China
Source: BMC Public Health. 2026 Mar 3;26:1157. doi: 10.1186/s12889-026-26766-x (PMC13064065; doi:10.1186/s12889-026-26766-x)
Supplement: Supplementary file 2 — Supplementary Material 2. [file 12889_2026_26766_MOESM2_ESM.docx]

**Survey on Parents’ Willingness and Influencing Factors Regarding School-Based Mental Health Screening for Junior High School Students**

Dear Parent,

Thank you very much for taking part in this survey.

The purpose of this study is to understand parents’ attitudes toward school-based mental health screening for junior high school students, in order to improve the implementation of mental health screening and provide scientific evidence for adolescent mental health services.

This questionnaire is anonymous and does not involve any personal or identifying information about you or your child. All data will be used solely for academic research, and we will strictly maintain the confidentiality of your responses.

Your participation will make a valuable contribution to the promotion of children’s mental health.

**Part 1 Personal Basic Information**

A1、The gender of your child:

□ 1... Male □ 2... Female

A2、The grade of your child:

□ 1... Grade 7 □ 2... Grade 8 □ 3... Grade 9

A3、Your year of birth: _______

A4、Your kinship with your child:

□ 1... Father □ 2... Mother

A5、Your educational background:

□ 1... Primary school or below □ 2... Junior high school □ 3... High school/ vocational school □ 4... Associate degree / bachelor's degree □ 5... Master's degree or above

A6、Your occupation:

□ 1... Unemployed / between jobs □ 2... Farmer/ herdsman/ fisher

□ 3... Manual worker (e.g., factory worker, construction worker, sanitation worker)

□ 4... Service worker (e.g., waiter/waitress, cook, driver, salesperson)

□ 5... Self-employed/ freelancer (e.g., blogger, tour guide, photographer)

□ 6... Clerical staff (e.g., office clerk, secretary, accountant, cashier, computer operator, statistician) □ 7... Professional/ technical personnel (e.g., researcher, teacher, engineer, doctor, lawyer, cultural worker) □ 8... Private business owner □ 9... Middle or senior manager in an enterprise

□ 10... Government or social administrator (e.g., senior official)

□ 11... Other (please specify: ______ )

A7、Your residential area:

□ 1 ...Urban □ 2... Rural

A8、Your family monthly income (RMB):

□ 1...＜5000 □ 2...5000-9999 □ 3...10000-19999

□ 4...20000-29999 □ 5...≥30000

A9、Your family structure：

□ 1... Intact family（child lives with the parents） □ 2... Single-parent family

□ 3... Stepfamily □ 4... Left-behind family（one or both parents are absent from home for an extended period of time） □ 5... Other (please specify: ______ )

A10、Your daily communication with your child (including forms such as face-to-face conversations and phone calls):

□ 1... Everyday □ 2... 4-6 times/week □ 3... 1-3 times/week

□ 4... Rare or almost no daily communication

A11、How would you describe your parenting style?

□ 1... Authoritative (high warmth × high control: clear and strict rules combined with emotional support)

□ 2... Authoritarian (low warmth × high control: emphasizes rules and obedience, with limited emotional expression)

□ 3... Permissive (low warmth × low control: little interference or guidance, few rules or demands)

□ 4... Indulgent (high warmth × low control: loving and caring, but sets few boundaries or rules)

□ 5... Uncertain

A12、How do you consider your relationship with your children?

□ 1... Very harmonious □ 2... Fairly harmonious □ 3... Average

□ 4... Not very harmonious □ 5... Very unharmonious

A13、The current overall academic performance ranking of your child in the class:

□ 1... Top tier (25%) □ 2... Upper middle tier (26%-50%)

□ 3... Lower middle tier (51%-75%) □ 4... Bottom tier (76%-100%)

A14、Has your child ever been diagnosed by a doctor with any mental or psychological disorder (such as depression, anxiety disorder, or attention deficit hyperactivity disorder)?

□ 1...Yes □ 2...No □ 3... Unsure

A15、Has your child ever participated in a school-based psychological screening or mental health questionnaire?

□ 1...Yes □ 2...No □ 3... Unsure

**Part 2 Understanding and Knowledge of Mental Health**

B1、How do you think the importance of mental health is for teenagers?

□ 1... Extremely important □ 2... Quite important □ 3... Neutral

□ 4... Not very important □ 5... Not important at all

B2、How severe do you think the current mental health issues among teenagers are?

□ 1... Extremely important □ 2... Quite important □ 3... Neutral

□ 4... Not very important □ 5... Not important at all □ 6... Unaware

B3、How well do you understand your child's psychological status?

□ 1... Very knowledgeable □ 2... Fairly knowledgeable □ 3... Average

□ 4... Limited knowledge □ 5... Unaware

B4、Have you noticed any of the following emotional or physical abnormalities in your child lasting for more than one week during the past three months? (You may select more than one option.)

□ 1... Persistent low mood □ 2... Loss of interest or pleasure □ 3... Sleep problems □ 4... Changes in appetite □ 5... Lack of energy/ fatigue □ 6... Attention or concentration problems □ 7... Social withdrawal □ 8... No obvious abnormalities

□ 9... Other (please specify: ______ )

B5、Does your child's school currently offer the following forms of mental health-related services? (You may select more than one option.)

□ 1... Presence of school mental health teachers / counselors □ 2... Mental health education classes offered □ 3... School psychological counseling room available

□ 4... Other (please specify: ______ ) □ 5...None □ 6...Unclear

B6、Through which channels do you usually obtain information or knowledge about mental health? (You may select more than one option.)

□ 1... Professional books or journals □ 2... School lectures or promotional activities

□ 3... Television or radio programs □ 4... Internet or social media

□ 5... Communication with family or friends □ 6... Communication with family or friends □ 7... Other (please specify: ______ ) □ 8... Unaware or unconcerned

**Part 3 Willingness of School-based Mental Health Screening for Children**

C1、Would you be willing to allow your child to participate in school-based mental health screening?

□ 1... Strongly willing □ 2... Somewhat willing □ 3... Uncertain

□ 4... Somewhat unwilling □ 5... strongly unwilling

C2、How willing are you to have your child undergo school-based screening for the following health topics?

(Please indicate your level of acceptance for each item on a scale of 1–5, where 1 = Completely unacceptable and 5 = Completely acceptable.)

1(Completely unacceptable) 2(Somewhat unacceptable) 3(Neutral) 4(Somewhat acceptable) 5(Completely acceptable）

C2_1 Sleep problems □ □ □ □ □

C2_2 Learning difficulties □ □ □ □ □

C2_3 Obesity □ □ □ □ □

C2_4 Myopia □ □ □ □ □

C2_5 Family relationships □ □ □ □ □

C2_6 Social media use □ □ □ □ □

C2_7 Internet addiction □ □ □ □ □

C2_8 School bullying □ □ □ □ □

C2_9 Anxiety symptoms □ □ □ □ □

C2_10 Depressive symptoms □ □ □ □ □

**Part 4 Concerns about School-based Mental Health Screening**

D1、To what extent do you agree with the following possible concerns about your child participating in mental health screening?

(Please indicate your level of agreement with each statement on a scale of 1–5, where 1 = strongly disagree and 5 = strongly agree.)

1(Strongly disagree) 2(Somewhat disagree) 3(Neutral) 4(Somewhat agree) 5(Strongly agree）

D1_1 Insufficient scientific validity and accuracy of screening tools □ □ □ □ □

D1_2 Children may not understand screening questions □ □ □ □ □

D1_3 Screening process may cause discomfort to children □ □ □ □ □

D1_4 Screening may not reflect true psychological status □ □ □ □ □

D1_5 Schools may lack professional screening capacity □ □ □ □ □

D1_6 Screening may take time away from academic studies □ □ □ □ □

D1_7 Questionnaire content may implicitly influence children □ □ □ □ □

D1_8 Risk of privacy breaches during screening □ □ □ □ □

D1_9 Screening may take time away from academic studies □ □ □ □ □

D1_10 Lack of post-screening support □ □ □ □ □

D1_11 Potential costs associated with screening and interventions □ □ □ □ □

**Part 5 Preferences for Screening Delivery and Result Feedback**

E1、How often do you think school-based mental health screenings should be conducted for middle school students?

□ 1... Once a month □ 2... Once per semester □ 3... Once per academic year

□ 4... Only once during middle school □ 5... Any of the above

□ 6... Screening is not necessary

E2、How long do you expect each school-based mental health screening to take?

□ 1... < 15 minutes □ 2... 15 minutes ≤ Time < 30 minutes □ 3... 30 minutes ≤ Time < 45 minutes □ 4... ≥ 45 minutes □ 5... Any duration is acceptable

E3、Who would you prefer to conduct school-based mental health screenings for your child? (You may select more than one option.)

□ 1... Homeroom teacher □ 2... School psychologist □ 3... School doctor

□ 4... External mental health professionals □ 5... Other (please specify: ______ )

E4、How would you prefer to receive your child’s mental health screening results？

□ 1... All parents receive feedback □ 2... Only parents of students at risk receive feedback

□ 3... Students decide whether to inform their parents □ 4... Other (please specify: ______ )

E5、What follow-up services do you think schools or health authorities should provide when students undergo mental health screening? (You may select more than one option.)

□ 1... Provide mental health education □ 2... Offer individual psychological counseling

□ 3... Referral to professional agencies □ 4... Home-school collaborative intervention

□ 5... Regular follow-up visits □ 6... No action required

□ 7... Other (please specify: ______ )

Thank you very much for taking the time to complete this questionnaire! Your responses provide valuable data to support our research on promoting students’ mental health.

We wish you all the best!
